# Dynamic taxonomy generation for future skills identification using a named entity recognition and relation extraction pipeline

**DOI:** 10.3389/frai.2025.1579998

**Published:** 2025-07-02

**Authors:** Luis Jose Gonzalez-Gomez, Sofia Margarita Hernandez-Munoz, Abiel Borja, Fernando A. Arana-Salas, Jose Daniel Azofeifa, Julieta Noguez, Patricia Caratozzolo

**Affiliations:** ^1^Institute for the Future of Education, Tecnologico de Monterrey, Monterrey, Mexico; ^2^School of Engineering and Sciences, Tecnologico de Monterrey, Mexico City, Mexico

**Keywords:** artificial intelligence, dynamic taxonomy, educational innovation, future skills, natural language processing, named entity recognition, professional development, word embeddings

## Abstract

**Introduction:**

The labor market is rapidly evolving, leading to a mismatch between existing Knowledge, Skills, and Abilities (KSAs) and future occupational requirements. Reports from organizations like the World Economic Forum and the OECD emphasize the need for dynamic skill identification. This paper introduces a novel system for constructing a dynamic taxonomy using Natural Language Processing (NLP) techniques, specifically Named Entity Recognition (NER) and Relation Extraction (RE), to identify and predict future skills. By leveraging machine learning models, this taxonomy aims to bridge the gap between current skills and future demands, contributing to educational and professional development.

**Methods:**

To achieve this, an NLP-based architecture was developed using a combination of text preprocessing, NER, and RE models. The NER model identifies and categorizes KSAs and occupations from a corpus of labor market reports, while the RE model establishes the relationships between these entities. A custom pipeline was used for PDF text extraction, tokenization, and lemmatization to standardize the data. The models were trained and evaluated using over 1,700 annotated documents, with the training process optimized for both entity recognition and relationship prediction accuracy.

**Results:**

The NER and RE models demonstrated promising performance. The NER model achieved a best micro-averaged F1-score of 65.38% in identifying occupations, skills, and knowledge entities. The RE model subsequently achieved a best micro-F1 score of 82.2% for accurately classifying semantic relationships between these entities at epoch 1,009. The taxonomy generated from these models effectively identified emerging skills and occupations, offering insights into future workforce requirements. Visualizations of the taxonomy were created using various graph structures, demonstrating its applicability across multiple sectors. The results indicate that this system can dynamically update and adapt to changes in skill demand over time.

**Discussion:**

The dynamic taxonomy model not only provides real-time updates on current competencies but also predicts emerging skill trends, offering a valuable tool for workforce planning. The high recall rates in NER suggest strong entity recognition capabilities, though precision improvements are needed to reduce false positives. Limitations include the need for a larger corpus and sector-specific models. Future work will focus on expanding the corpus, improving model accuracy, and incorporating expert feedback to further refine the taxonomy.

## 1 Introduction

Natural Language Processing (NLP) integrates linguistic principles, computer science, and artificial intelligence (AI) to emulate and understand human language using computational systems. Its primary aim is to facilitate a symbiotic relationship between computers and human language, enabling the understanding of extensive textual data, including its contextual complexities (Li et al., 2022). As NLP evolves, its applications have expanded beyond basic language understanding to more sophisticated ones, such as sentiment analysis, topic extraction, entity recognition and relation extraction, to name a few. These advancements are supported by the synergy between NLP techniques and machine learning algorithms (Li et al., 2023).

But NLP is not the only thing that has evolved dramatically lately. The labor market is also undergoing rapid transformations that demand an innovative approach to competences identification. And here is where the two meet: NLP tools such as entity recognition and relationship extraction models can be used for the creation of taxonomies that identify competences or Knowledge, Skills, and Abilities (KSA). These taxonomies would reflect the current state of demanded KSA extracted from the data sources they were applied to. Dynamic KSA taxonomies could also act as an occupational profiling predictive framework for the changing labor market by taking advantage of the predictive capabilities offered by NLP models.

This research aims to contribute to the development of a forward-looking approach to identifying KSA, ensuring adaptability in the face of rapid technological and industrial changes. By anticipating future skill demands, this approach could support the creation of dynamic, responsive taxonomies that can be continuously updated in real-time.

In the following sections, we explore different NLP tools such as NER and RE for the detection, extraction, and classification of competences. Through this exploration, we aim to exploit the potential of these tools for constructing a dynamic taxonomy that not only identifies current skills but, more critically, anticipates and categorizes the skills that will shape the workforce of the future.

In our approach, NER detects and classifies occupational and competence-related terms from unstructured labor market data. RE then establishes semantic links between these entities, enabling the dynamic generation and updating of taxonomic relationships. This pipeline is central to building a predictive, evolving taxonomy that reflects the changing nature of workforce demands.

The key contributions of this study are as follows:

- We present an NLP-based architecture that integrates NER and RE to automatically build and update a dynamic Knowledge, Skills, and Abilities (KSAs) taxonomy.
- We construct and annotate a high-quality corpus of over 1,700 labor market documents for training and evaluation.
- We demonstrate the effectiveness of our models with detailed performance metrics and visualize the resulting taxonomies across domains.
- We introduce a lightweight, scalable pipeline for extracting skills and occupations from PDF-based reports using custom text pre-processing.

Although common preprocessing techniques such as PDF parsing, tokenization, and lemmatization are employed, our innovation lies in their orchestration within a framework that supports real-time taxonomy updates and domain adaptation.

## 2 State of the art

NLP has undergone a significant transformation over the past decade, moving from basic text analysis to complex semantic modeling. Early work explored foundational capabilities such as entity recognition, classification, and sentiment analysis, laying the groundwork for extracting meaningful patterns from unstructured text (Afshar et al., 2019; Blandin et al., 2020; Dehbozorgi and Mohandoss, 2021).

As NLP matured, researchers introduced interdisciplinary approaches that integrated psychological, educational, and structural perspectives. For instance, Cao et al. (2022) focused on user identity in sentiment analysis for personalized feedback, and Laverghetta Jr. et al. (2021) investigated psychometric prediction from language models. These studies contributed to broadening NLP's scope beyond basic information extraction toward richer interpretative tasks.

However, the field has more recently pivoted toward structuring and forecasting professional skills, using taxonomies and knowledge graphs to make sense of workforce data. This trajectory is highly relevant to our objective of building a dynamic taxonomy for future skills.

Jose Gonzalez-Gomez et al. (2024) conducted a systematic review on NLP for skill acquisition, emphasizing a trend toward identifying domain-specific competences using relation-aware models. Building on this, three particularly relevant studies are:

- Huang et al. (2020) introduced CoRel, a framework for seed-guided topical taxonomy construction that integrates relation transferring and concept learning. Their method enriches taxonomy nodes with both semantic depth and structural flexibility.
- Jiang et al. (2022) developed TaxoEnrich, a self-supervised method for completing taxonomies using structure-semantic embeddings, outperforming traditional enrichment methods through dual-encoder architectures.
- Lin et al. (2023) focused on skill graph construction by generating synthetic text pairs and training language models to identify semantic links between skills, with practical applications in LinkedIn's talent platform.

While these approaches laid critical groundwork, our method introduces several innovations:

- Domain-specific focus: prior frameworks are general-purpose or platform-specific. In contrast, our approach targets labor market intelligence by modeling KSAs extracted from high-level policy and industry documents.
- NER + RE integration: we combine Named Entity Recognition and Relation Extraction into a single architecture to detect, classify, and connect competences and occupations.
- Real-time update capabilities: unlike static or seed-expansion models, our taxonomy evolves automatically with new documents, adapting to labor market shifts.
- Interactive visualization and application: beyond taxonomy building, we deliver dynamic visualizations that can serve as a guide to educators, institutions, and professionals in upskilling and reskilling strategies.

In summary, this work aligns with recent efforts in dynamic taxonomy generation but extends them by providing a domain-specific, adaptive, and visualization-ready solution tailored to emerging workforce needs.

## 3 Methods and process

To effectively articulate the methodology behind our research, we structured this section to delineate the systematic steps undertaken to achieve our objectives. This section outlines the innovative techniques and computational strategies employed to develop a dynamic taxonomy framework based on NER and RE. Our approach integrates comprehensive pre-processing of labor market data, model training on annotated datasets, and predictive modeling to identify and classify KSAs. By leveraging state-of-the-art tools and techniques in NLP, including advanced text cleaning, annotation workflows, and transformer-based architectures, this section provides a detailed blueprint of the methods utilized to ensure accuracy, adaptability, and relevance in addressing the dynamic needs of the future workforce.

### 3.1 Research goals and contributions

The primary objective of this research is to develop a dynamic taxonomy framework that leverages NLP techniques, specifically NER and RE, to identify and predict future KSAs required in the evolving labor market. This taxonomy is designed to support real-time updates, adapt to changes in skill demand, and inform workforce planning strategies across sectors.

To this end, our approach is guided by the following research questions:

1. **How can NER and RE models be applied to identify and classify emerging competencies in the labor market?**
  We explore how NLP tools, particularly NER and RE, can be trained to extract KSAs from unstructured textual data, such as labor market reports and job postings.
2. **What is the role of a dynamic KSA taxonomy in predicting future workforce competencies?**
  We examine how a continuously evolving taxonomy can be used as a forward-looking tool to anticipate changes in occupational requirements and emerging trends.
3. **How effective is the proposed NLP-based architecture in achieving high accuracy for both entity recognition and relationship prediction?**
  We evaluate the performance of our NER and RE models using precision, recall, and F1-score metrics, and assess their potential for generalization and scalability.

#### 3.1.1 Contributions

This study introduces several key contributions:

- A domain-specific architecture for dynamic taxonomy construction, combining NER and RE to extract and semantically link KSAs and occupations from large volumes of labor market texts.
- A custom annotation corpus of over 1,700 labor market documents, manually labeled with occupations, skills, and knowledge entities, and used to train and evaluate the models.
- The integration of a hybrid embedding strategy, combining the tok2vec architecture (based on character-level and hash embeddings) with transformer-based models (RoBERTa) to improve entity recognition accuracy and relationship prediction in noisy real-world data.
- A lightweight NLP pipeline that includes PDF parsing, advanced text preprocessing, and linguistic normalization steps to clean and standardize the raw data.
- Interactive visualizations that illustrate the relationships among occupations and KSAs, supporting real-world applications in reskilling, curriculum design, and labor market analytics.

### 3.2 Text pre-processing

**Figure 1:**
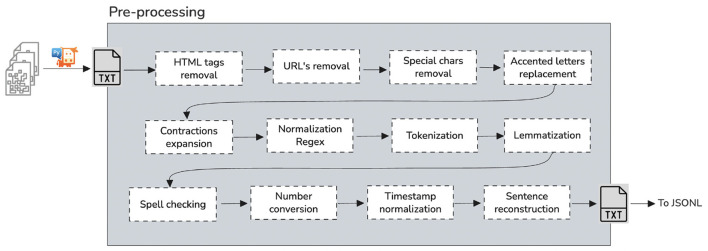
Pre-processing.

Text pre-processing (Figure 1) is traditionally an important step for natural language processing (NLP) tasks. It transforms text into a more digestible form so that machine learning algorithms can perform better (Zaman et al., 2024). The corpus we employ is structured in Portable Document Format (PDF), encompassing vector graphics, text, and bitmap graphics. Our primary objective is to retrieve text from PDF files. In PDF, text is delineated through text elements embedded in page content streams. Each text element designates the positions at which characters should be rendered. The characters are defined using the encoding of a designated font resource. Consequently, text in PDF is not encoded in a straightforward, plain-text format.

We found that our corpus PDFs may also encompass content such as Vector graphics, comprising shapes and lines, for illustrations and designs. Also, we found that our corpus contained raster graphics for photographs and various image types.

Now, the corpus PDF incorporates three main technologies. First, a comparable subset of the PostScript page description programming language is presented in a declarative format for generating layout and graphics. Second, a font-embedding/replacement system facilitates the embedding of fonts within documents. Third, A structured storage system that consolidates these elements and any associated content into a single file, with data compression where applicable. For us to maintain a pure text extraction from our PDF files, we employed PyMuPDF for text extraction. PyMuPDF is a Python library known for its high performance in extracting, analyzing, converting, and manipulating data within PDF (and other) documents.

### 3.3 PDF parsing and text cleaning

Before any downstream NLP processing, the corpus needs to undergo structured parsing and cleaning to be converted into high-quality, machine-readable text.

PDF documents in our corpus consist of text elements, vector graphics, and raster images. Parsing such documents requires interpreting text elements embedded in page content streams, which are not readily extractable as plain text. To address this, we used PyMuPDF, a high-performance Python library for extracting and manipulating document data.

After parsing, a comprehensive text cleaning pipeline was applied. This included removing HTML tags, URLs, special characters, emojis, accented characters, and expanding contractions. We used a combination of regex patterns and Python libraries like re, unidecode, and spacy to normalize the text.

We also integrated tokenization and lemmatization into this pipeline. Using NLTK's word_tokenize and WordNetLemmatizer, each sentence was split into tokens and reduced to base forms, allowing for consistent entity recognition in downstream modeling. These steps significantly improve linguistic uniformity and reduce noise in the training data.

Additional steps included spell-checking, the conversion of numbers to text, timestamp normalization, and sentence reconstruction based on punctuation. The final cleaned and tokenized dataset was exported in JSONL format, supporting efficient streaming annotation and training using Prodigy.

### 3.4 Corpus creation overview

**Figure 2:**
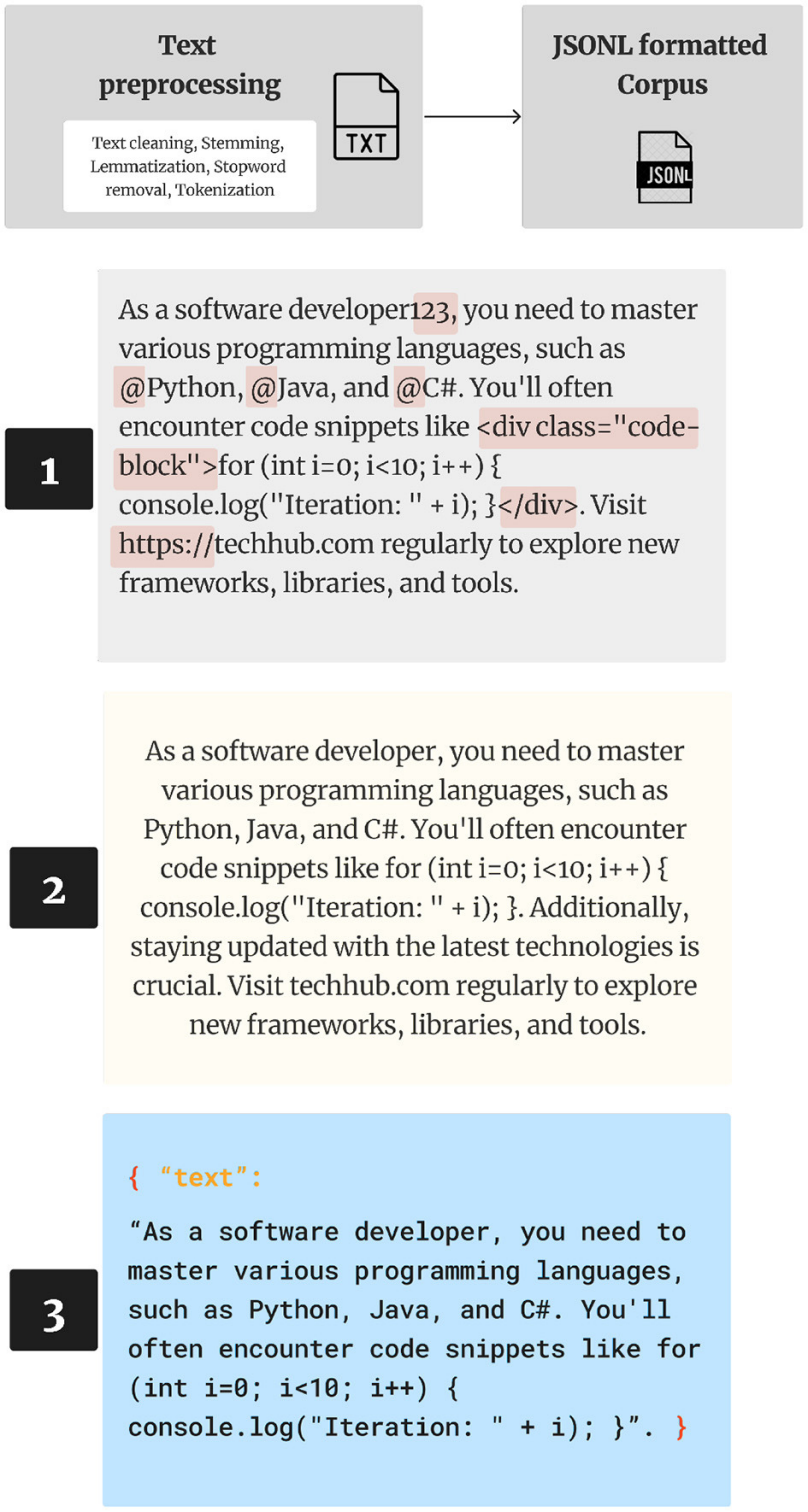
Corpus creation process.

JSON Lines text format, also called newline-delimited JSON. JSON Lines is a convenient format for storing structured data that may be processed one record at a time. The final corpus was exported as a JSONL, denoted by the .jsonl file extension. JSONL is a text-based format that closely resembles the JSON format but employs newline characters to distinguish between individual JSON values. We used JSONL file extension because the annotation tool we employed, called Prodigy, uses it as the main format for dynamic parsing, and as we will see, this approach proves advantageous when dealing with exceptionally large files on machines with restricted memory resources (Figure 2).

There are three notable distinctions between JSON and JSONL formats:

1. JSONL utilizes UTF-8 encoding, in contrast to JSON, which permits the encoding of Unicode strings through ASCII escape sequences.
2. Each line in JSONL constitutes a valid JSON value.
3. Lines are separated by newline characters (“\n").

After defining our architectural approach for NER and RE, we implemented training workflows using the Entity Recognizer class from spaCy, which allows for fine-tuning on custom datasets. To support this, we created annotated examples that defined the named entities and relational labels required by the models. Annotation was conducted manually using an interactive tool that allowed for real-time customization of entity spans and relation types. The annotation interface also aligned with spaCy's native tokenization system, ensuring that entity boundaries remained consistent across the training corpus and downstream parsing tasks. This process provided a controlled and repeatable foundation for the NER and RE models used in the dynamic taxonomy construction.

### 3.5 NER labeling

**Figure 3:**
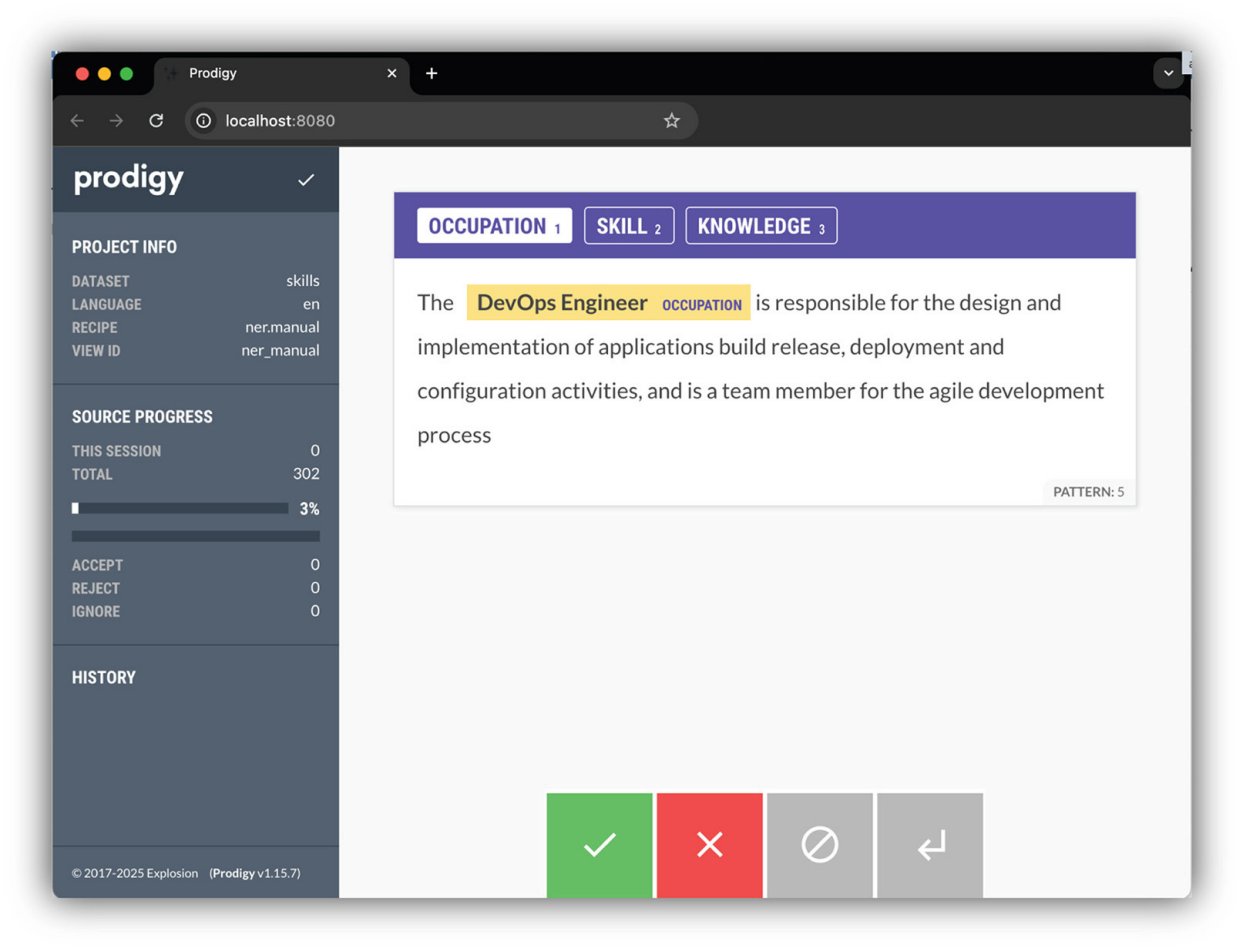
Annotation process.

NER extracts information from text (Liang et al., 2019). In our research endeavor, we employed the ner.manual interface within Prodigy recipes (Figure 3). This interface facilitated the manual annotation of KSAs and occupations, predefining them as labels for a more focused analysis. The resultant annotations, capturing entity spans denoting specific information in the text, were meticulously preserved in a PostgreSQL database. This systematic recording of marked annotations serves as a foundational dataset for our NER model refinement.

All annotations were performed manually by the research team with advanced knowledge of occupational classification and NLP techniques. To reduce potential bias, we incorporated multiple review rounds and cross-validation across annotators. Future work may include engaging independent domain experts for annotation validation.

### 3.6 NER model

**Figure 4:**
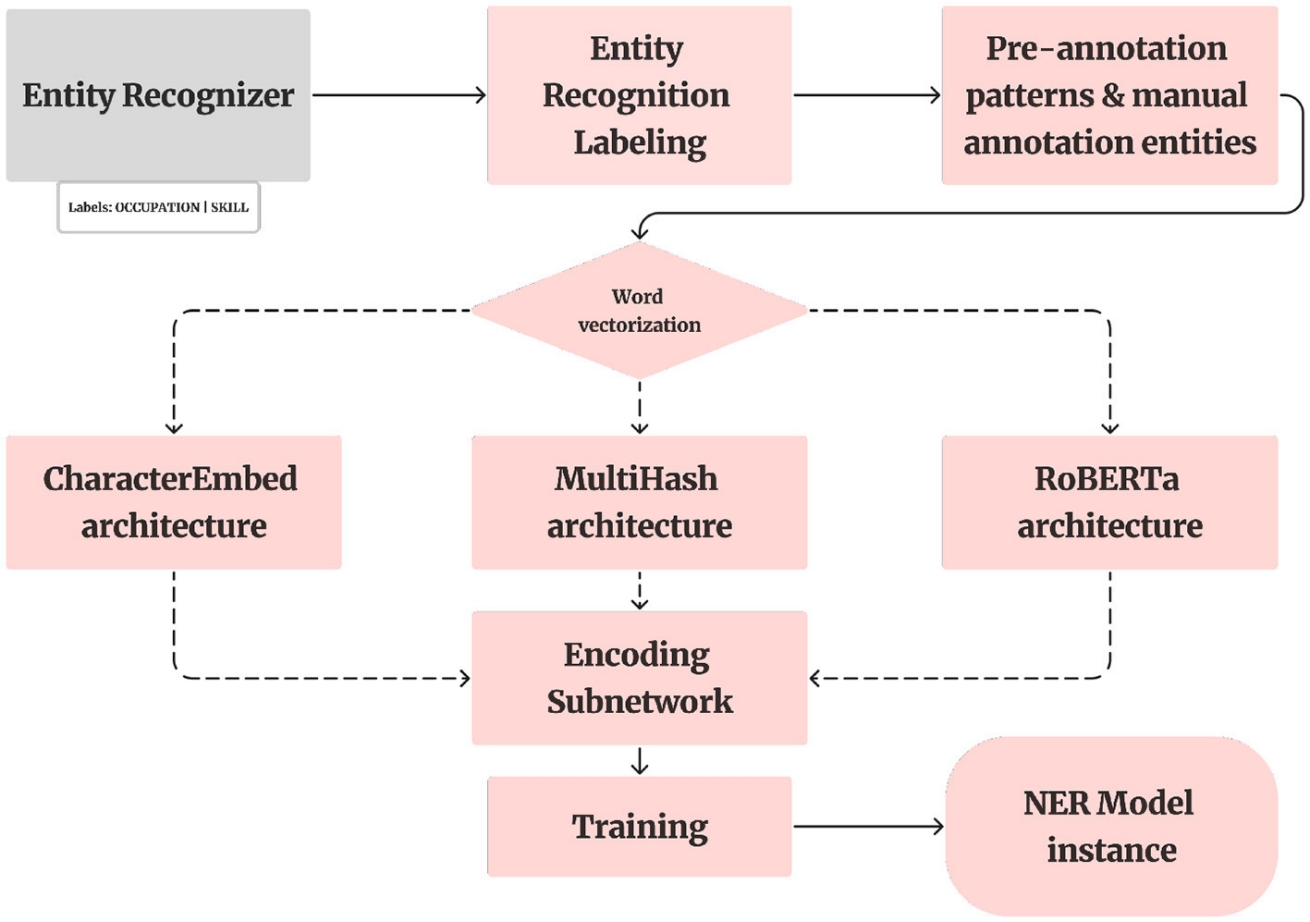
NER model.

This section details the NER model based on a custom tok2vec pipeline, which combines character embeddings and contextual vectorization for optimal accuracy. Following the creation of our annotated dataset, we utilized a pre-trained spaCy model (Figure 4). This model, optimized for CPU, encompasses a comprehensive pipeline with components such as a word vectorizer, tagger, parser, senter, ner, attribute_ruler, and lemmatizer.

The application of the spaCy Entity Recognizer component was pivotal in augmenting our annotated training data. Leveraging this component, we systematically processed each document, introducing entity annotations for our custom entity types, namely skills, occupation roles, knowledge, and abilities.

The NER model in spaCy employs a machine-learning algorithm called a conditional random field (CRF). This model processes a sequence of words (tokens) as its input and produces a corresponding sequence of labels. These labels denote whether each token is associated with a named entity and, if so, specify the type of entity to which it belongs (Jurafsky and Martin, 2009).

The spaCy Entity Recognizer class functions as a component for transition-based entity recognition, employing transition-based parsing. Transition-based parsing is a method for structured prediction, framing the prediction of structure as a sequence of state transitions. In this scenario, the prediction model for neural network state involves either two or three subnetworks.

Regarding the first subnetwork, we adopt a classical approach commonly employed in information retrieval tasks within Natural Language Processing. Initially, we utilize a tok2vec architecture, wherein each token is mapped to a vector representation. To accomplish this, we leverage both the built-in CharacterEmbed architecture and the MultiHash embed architecture provided by spaCy. The two results yielded different tok2vec loss values, as we will present them in the next section.

There are several well-established approaches for word vectorization. For instance, word2vec learns continuous vector representations of words based on context. GloVe constructs word vectors by analyzing global co-occurrence statistics. fastText extends Word2Vec by representing each word as a bag of character n-grams. Transformer-based models, such as BERT (Bidirectional Encoder Representations from Transformers) and ELMo (Embeddings from Language Models), have also proven effective.

Following the tok2vec approach, we employed the RoBERTa transformer model, which resulted in even more favorable loss values (as detailed in the next section). This improvement can be attributed to the use of attention mechanisms, allowing the model to consider the entire context of a word bidirectionally. In contrast, shallow neural network architectures like tok2vec use a simpler structure, focusing on subword information. In summary, the critical distinction lies in context sensitivity: RoBERTa produces embeddings sensitive to context, while FastText generates embeddings independent of context (Brauwers and Frasincar, 2023).

Although RoBERTa embeddings handle out-of-vocabulary tokens via byte-pair encoding, preprocessing such as punctuation removal and character normalization was retained to align with downstream token matching in annotation and visualization modules. The entity spans predicted by the NER model serve as the input pairs for the Relation Extraction phase, forming a tightly integrated pipeline. Accurate entity detection is thus foundational for the effectiveness of relation prediction.

### 3.7 RE

**Figure 5:**
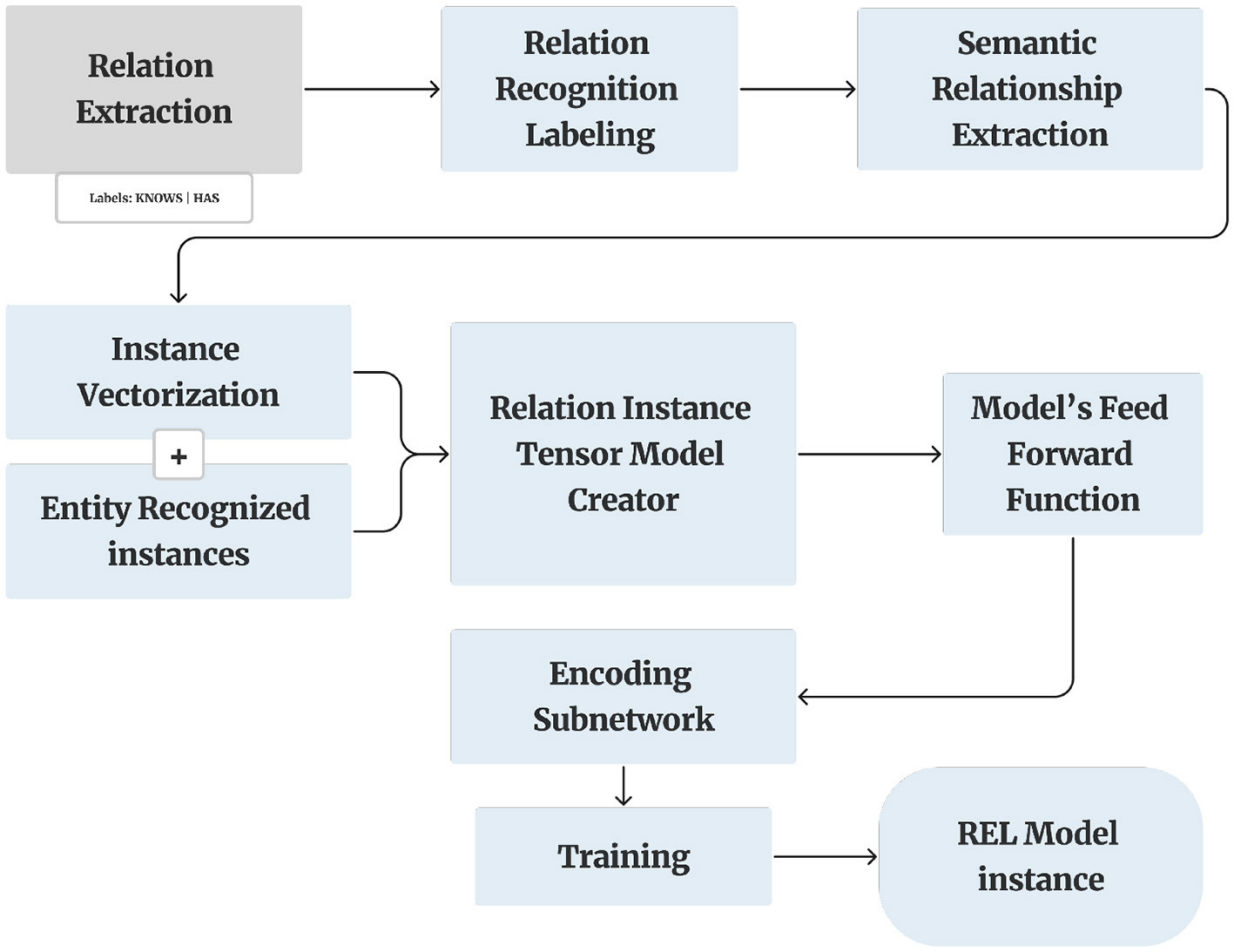
Relation extraction model.

RE is the task of predicting attributes and relations for entities in a sentence (Huang and Wang, 2017). RE is a crucial process for analyzing structured text data (Figure 5). RE involves identifying and categorizing semantic relationships between named entities within the text. This task is fundamental in understanding the complex interactions and associations present in the data. Once the entities are identified, the next step is to determine the relationships between these entities. This is done by analyzing the context in which the entities co-occur, paying close attention to the linguistic and syntactic cues that may indicate a particular type of relationship. To accomplish this, we employ Prodigy, whose interactive and user-friendly interface significantly enhances the efficiency and accuracy of the labeling process.

**Figure 6:**
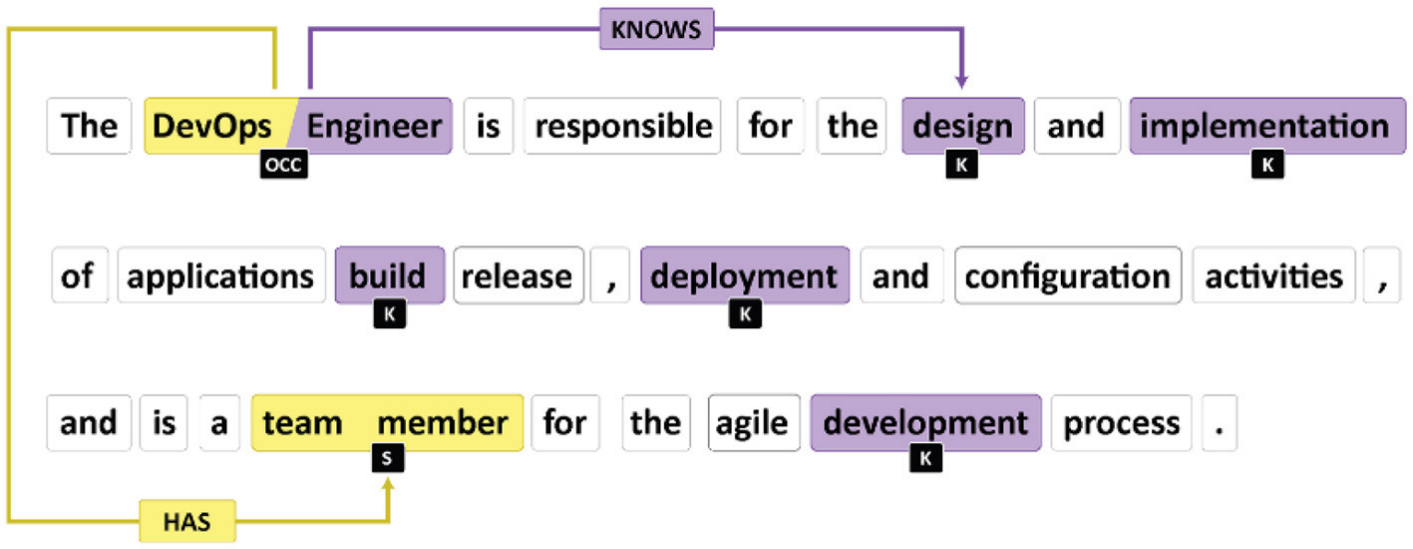
Relation extraction labeling.

In the context of RE, Prodigy facilitates the identification and categorization of semantic relationships between entities within sentences. This involves annotating pairs of words or phrases that hold a specific semantic connection, such as “has” or “knows” (Figure 6). By leveraging Prodigy, we can efficiently train a model to recognize and predict these relationships in new, unseen texts.

To enhance the accuracy and depth of our RE analysis, we integrate both manual and automated methods. The manual method involves human annotators who meticulously review the text and label the relationships based on their understanding and interpretation of the context. This approach, while time-consuming, provides high accuracy and is invaluable in establishing an initial training set for automated methods.

On the automated front, we leverage machine learning algorithms that are trained on the manually annotated data. These algorithms are designed to learn from the patterns in the labeled examples and can then be used to identify and categorize relationships in new, unseen text. The use of machine learning not only scales up the RE process but also brings in a level of consistency and efficiency that is challenging to achieve with manual methods alone.

### 3.8 Model training and evaluation

This section presents the training methodology, learning architectures, hyperparameters, and evaluation processes for the two main components of our dynamic taxonomy framework: the NER model and the RE model. Each model was trained independently on an annotated corpus and evaluated using standard classification metrics.

### 3.9 Overview of models and learning architectures

Two models were developed and trained:

- NER: implemented using spaCy's transition-based parser with a tok2vec encoder that integrates hash embeddings and character-level CNNs. This model was responsible for identifying and classifying entities such as occupations and KSAs from unstructured labor market texts.
- RE: implemented using a span-based classification architecture with RoBERTa-base for the embeddings via the spacy-transformers library. This model predicts semantic relationships between pairs of recognized entities based on contextual cues.

Each model was trained on a custom annotated dataset of 1,739 labor market reports prepared using the Prodigy annotation tool.

#### 3.9.1 NER model configuration

The NER model was built using spaCy's tok2vec encoder combined with a transition-based parser. The encoder constructs token-level representations through a hybrid of subword and character-level embeddings. These representations were used to classify spans into predefined entity types.

The model training followed a learning rate schedule with early stopping based on the F1-score of a held-out development set.

#### 3.9.2 RE model configuration

The RE model identifies relationships between entity pairs using a span-based classifier on top of RoBERTa contextual embeddings. To handle long input documents, a sliding span window approach was used to retain relational context within text segments.

To reduce noise from non-informative span pairs, we applied *null_annotation_setter* to filter out irrelevant relations during preprocessing. Stratified batch sampling was used to ensure balanced learning across rare and frequent relation types. The training configuration for the model can be found in Table 1.

**Table 1:** NER model hyperparameters.

**Parameter**	**Value**
Learning algorithm	Transition-based parser
Embedding method	tok2vec (HashEmbedCNN + CharCNN)
Batch size	1,000
Random seed	342
Maxout width	128
Dropout	0.2
Activation function	ReLU
Optimizer	Adam
Epochs	30 (early stopping after 20)

#### 3.9.3 Training workflow

The model training pipeline consisted of four main stages:

1. **Data preparation:** Prodigy-annotated data were exported in JSONL format and preprocessed for token alignment, span indexing, and sentence segmentation.
2. **Pipeline configuration:** Independent configuration files (.cfg and .yaml) defined the NER and RE pipeline architectures, enabling modular training with reusable settings.
3. **Model training:** NER and RE models were trained separately using GPU acceleration for the RE model. Loss, precision, recall, and F1-score were tracked after each epoch, and best-performing checkpoints were retained.
4. **Evaluation:** Final model performance was measured on a validation set using micro-averaged metrics. These results are analyzed in Section 4.

Although the architecture was developed and tested using English-language reports, the methodology can be adapted to other languages by adjusting the tokenization, embeddings, and annotation schema to the target linguistic context. Future work could include testing multilingual capabilities for broader applicability.

#### 3.9.4 Evaluation metrics

Evaluation focused on micro-averaged precision, recall, and F1-score, measured across both entity and relation classes. The NER model was trained for 30 epochs and showed best performance around epoch 22, indicating relatively stable convergence through the full training window. The RE model, which was trained using a span-based RoBERTa classifier, ran for 10,000 steps and achieved its highest F1 scores between step 2,400 and 2,600, after which performance plateaued. Early stopping and checkpointing were used in both pipelines to retain the best-performing models for downstream inference. It is important to note:

- Early stopping was used to select the optimal model checkpoint.
- Extended training was conducted for analysis purposes only, to assess performance trends and ensure model stability.

#### 3.9.5 Hardware and runtime environment

All training and evaluation were conducted on a highperformance computing system. See Table 2 for the model training hyper parameters and Table 3 for the specifications.

**Table 2:** RE model hyperparameters.

**Parameter**	**Value**
Learning algorithm	Span-based classifier
Base model	RoBERTa-base
Span window size	128
Span stride	96
Batch size	4096
Tokenizer	use_fast = True
Span getter	strided_spans
Dropout	0.1
Optimizer	AdamW
Epochs	10

**Table 3:** Hardware and runtime environment.

**Component**	**Specification**
GPU	NVIDIA RTX 3060 (12 GB VRAM)
CPU	AMD Ryzen 5 5600, 6-core @ 3.5 GHz
RAM	32 GB DDR4
Storage	1 TB NVMe SSD
Operating system	Ubuntu 22.04 LTS
Python environment	Python 3.10
spaCy version	v3.5
Transformer library	spacy-transformers
Deep learning backend	PyTorch with CUDA 11.8

This setup provided sufficient memory and GPU compute to efficiently train both the NER and RE models, particularly for transformer-based embedding tasks.

The overall architecture of the Relation Recognizer methodology, presented in Figure 5, shows the end-to-end flow from document input through entity recognition and relation classification. While the diagram focuses on the conceptual NLP pipeline and does not include implementation-specific details such as the sliding span windowing strategy used in training, it effectively conveys the model's core components as described earlier.

This modular structure was intentionally designed to support future modifications and extensions–such as integrating alternative entity taxonomies, expanding classification types, or applying transfer learning to adjacent labor market domains. Although the architecture was developed and tested using English-language reports, the methodology can be adapted to other languages by adjusting the tokenization, embeddings, and annotation schema to the target linguistic context. Future work could include testing multilingual capabilities for broader applicability.

With the models trained, optimized, and validated, the following section presents a performance analysis of both the NER and RE components, demonstrating their effectiveness in extracting meaningful occupational and skill relationships from real-world labor market data.

## 4 Results and analysis

This section presents the performance evaluation of the proposed NER and RE models and analyzes the outputs generated by the dynamic taxonomy system. Table 4 summarizes the number of labeled entities for KSAs annotated in our dataset across the 1,739 documents used for model training and evaluation. This distribution reflects the relative frequency of each KSA category in the labor reports.

**Table 4:** Distribution of annotated KSAs in the training corpus.

**Entity type**	**Count**
Knowledge	2,345
Skills	3,125
Abilities	1,967
**Total**	7,437

### 4.1 NER model training performance

Table 5 shows the NER model's training performance measured at various epochs. Epoch 0 represents the untrained model baseline with negligible scores.

**Table 5:** Performance metrics of the NER model across training phases.

**Epoch**	**Step**	**Tok2Vec loss**	**NER loss**	**Micro F1**	**Precision**	**Recall**	**Score**
0	0	0.00	76.50	0.00	0.00	0.00	0.00
3	200	59.88	1593.18	60.87	82.35	48.28	0.61
6	400	63.12	256.86	52.17	70.59	41.38	0.52
9	600	101.58	187.52	56.00	66.67	48.28	0.56
12	800	110.26	115.67	57.14	59.26	55.17	0.57
16	1000	95.46	97.21	54.84	51.52	58.62	0.55
19	1200	60.58	75.62	**65.38**	73.91	58.62	0.65
22	1400	94.80	73.64	60.00	71.43	51.72	0.60
25	1600	104.52	85.98	55.17	55.17	55.17	0.55
29	1800	60.69	70.00	57.14	70.00	48.28	0.57
32	2000	94.32	74.03	49.06	54.17	44.83	0.49
35	2200	53.75	58.85	49.12	50.00	48.28	0.49
38	2400	176.12	75.86	52.17	70.59	41.38	0.52
41	2600	140.49	77.40	57.14	59.26	55.17	0.57
45	2800	29.61	54.59	53.57	55.56	51.72	0.54

The bold value indicates Highest Micro F1 value.

The best F1-score of 65.38% occurs at epoch 19, showing effective training progress. Loss metrics indicate steady reduction, and subsequent epochs show slight performance fluctuations typical of deep sequence models.

### 4.2 RE model training performance

Table 6 reports RE training metrics at selected steps. Initial step 0 shows very low baseline performance.

**Table 6:** Training performance of the RE model over selected phases.

**Epoch**	**Steps**	**Loss**	**Precision**	**Recall**	**Micro F1**	**SCORE**
0	0	0.41	6.37	32.24	10.64	0.11
18	500	7.02	81.88	80.26	81.06	0.81
37	1000	0.93	76.05	83.55	79.62	0.80
55	1500	0.79	81.51	78.29	79.87	0.80
74	2000	0.52	77.36	80.92	79.10	0.79
95	2500	0.19	73.99	84.21	78.77	0.79
136	3000	0.07	76.25	80.26	78.21	0.78
209	3500	0.45	78.40	83.55	80.89	0.81
309	4000	0.01	78.75	82.89	80.77	0.81
409	4500	0.00	78.75	82.89	80.77	0.81
509	5000	0.06	80.00	84.21	82.05	0.82
609	5500	0.01	79.75	82.89	81.29	0.81
709	6000	0.01	79.87	83.55	81.67	0.82
809	6500	0.00	80.77	82.89	81.82	0.82
909	7000	0.00	79.87	83.55	81.67	0.82
1009	7500	0.00	80.89	83.55	**82.20**	0.82
1109	8000	0.00	79.87	83.55	81.67	0.82
1209	8500	0.00	79.38	83.55	81.41	0.81
1309	9000	0.17	78.12	82.24	80.13	0.80
1409	9500	0.03	77.63	77.63	77.63	0.78
1509	10000	0.00	77.27	78.29	77.78	0.78

The bold value indicates Highest Micro F1 value.

The best F1-score of 82.20% at update 7500 (epoch 1009) indicates strong relation extraction performance. Loss metrics steadily decrease, confirming convergence. The micro-F1 score was used to evaluate RE performance due to its robustness in handling imbalanced class distributions, which are common in relation extraction tasks.

### 4.3 RE model evaluation

Table 7 reports relation extraction evaluation across different confidence thresholds, displaying precision, recall, and F1-score. While the best validation performance was 82.2% (micro-F1), the maximum F1-score under threshold-based classification occurred at threshold 0.50 with 74.90%. This reflects the trade-off between confidence thresholds and overall relation prediction performance.

**Table 7:** Evaluation of the relation extraction model.

**Threshold**	**Precision**	**Recall**	**Micro F1**
0.00	2.59	100.00	5.04
0.05	65.56	81.15	72.53
0.10	67.36	79.51	72.93
0.20	68.84	77.87	73.08
0.30	70.15	77.05	73.44
0.40	70.68	77.05	73.73
0.50	72.87	77.05	**74.90**
0.60	73.81	76.23	75.00
0.70	75.00	76.23	75.61
0.80	76.23	76.23	76.23
0.90	79.31	75.41	77.31
0.99	81.00	66.39	72.97
1.00	88.00	54.10	67.01

The bold value indicates Micro F1 value for threshold at 0.50.

**Figure 7:**
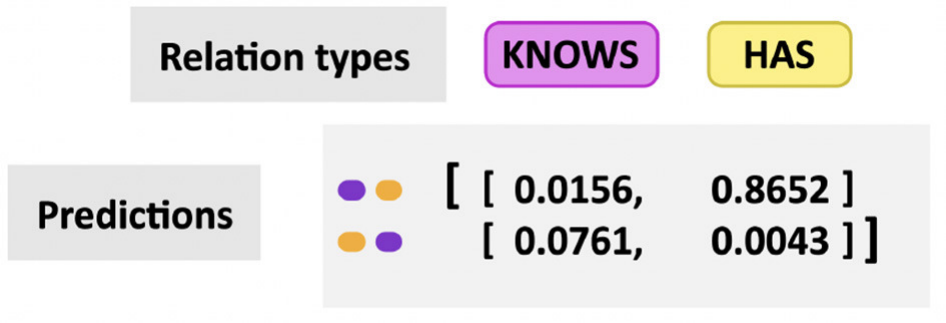
Sample output matrix showing predicted relationships between entity pairs.

The output from the RE model is a matrix holding on each row a combination of two found entities and their corresponding value for each of the relation verbs used (an example in Figure 7). Since the relation between the two entities can be found in any direction, two rows are needed for each pair of entities, changing the order on each row.

**Figure 8:**
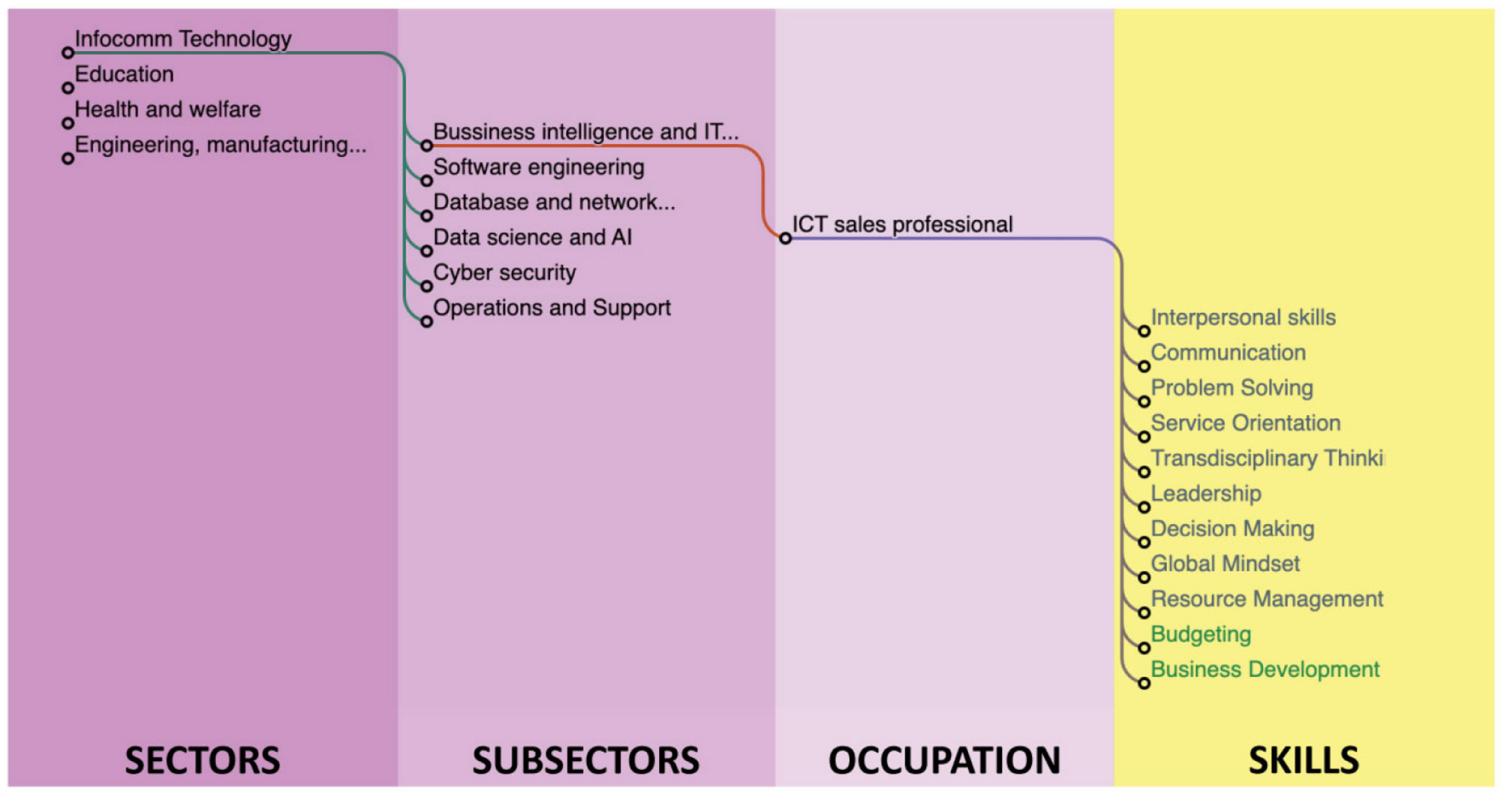
A dendogram diagram with hierarchical representation.

**Figure 9:**
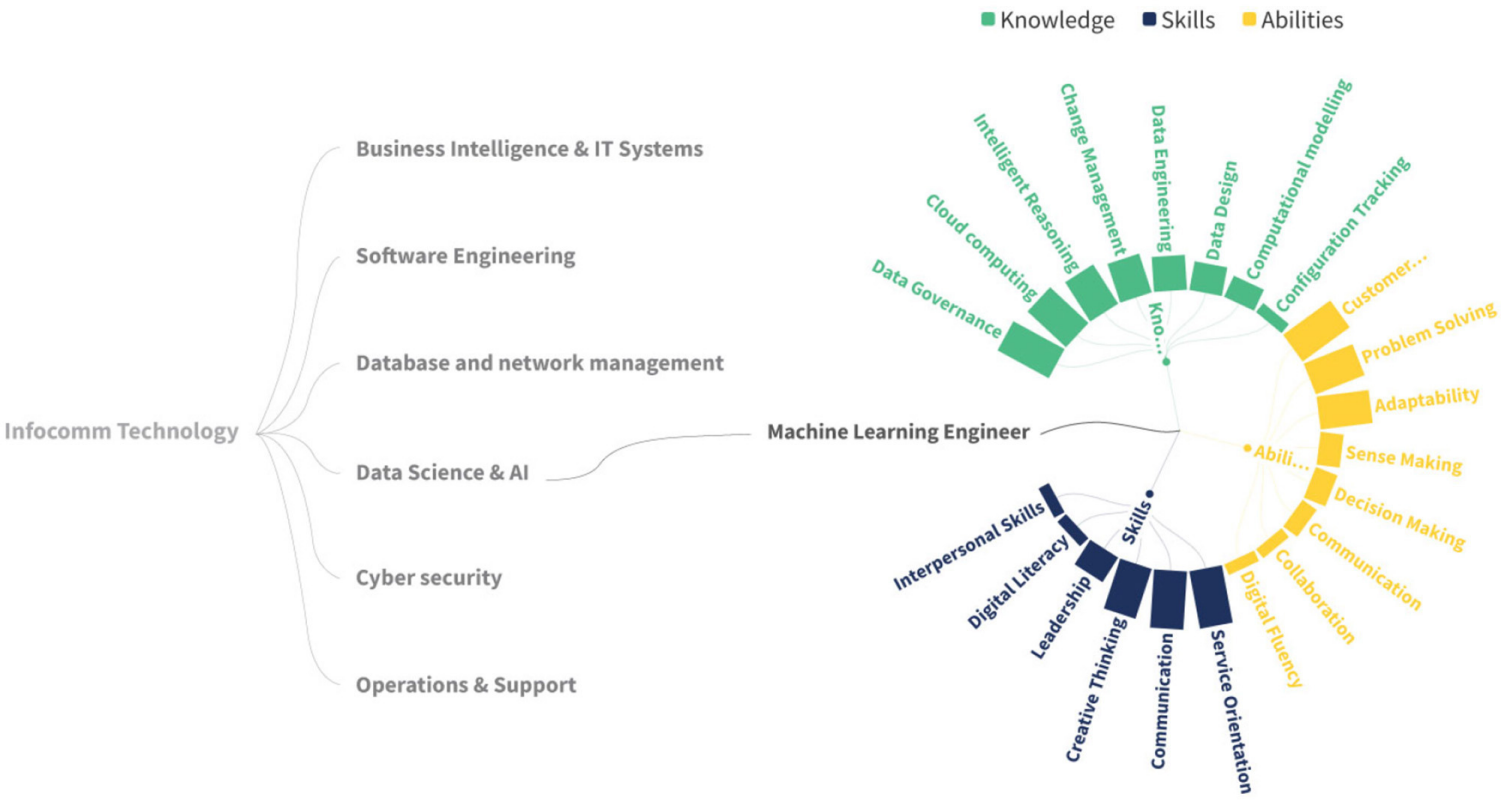
A Radial chart with KSA's.

The taxonomy relations were stored in a relational database, which was accessed via an HTTP API to generate the required input for visualizations. To visualize the initial taxonomy, we created some prototypes to test various options using the seed data. Two of these prototypes are showcased in Figures 8, 9.

**Figure 10:**
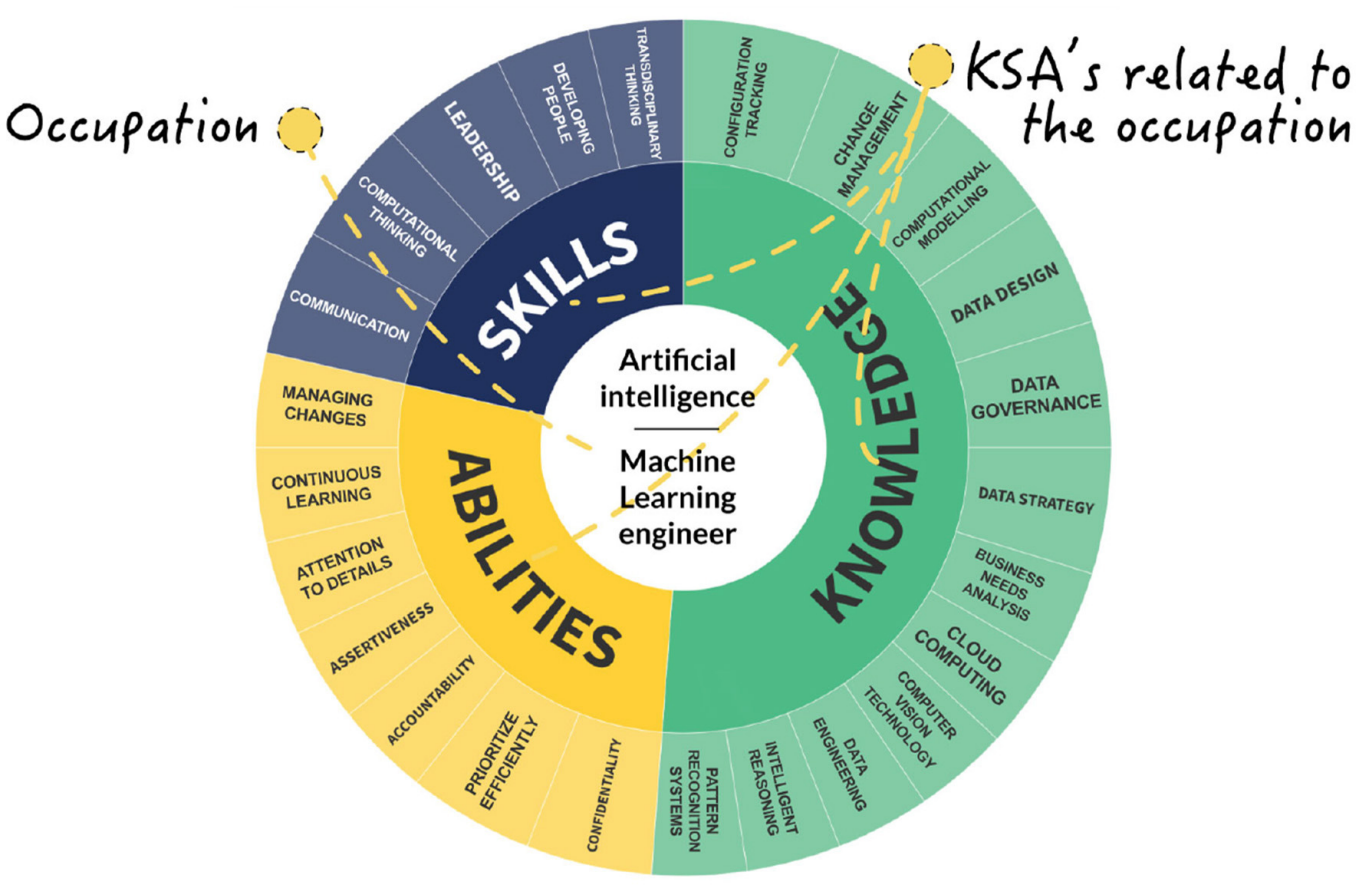
Single occupation and KSA's view.

As the architecture is independent of any specific library, various prototypes of web plotting charts were examined to showcase its versatility. Figure 10 shows a chart implemented with data from the INFOCOMM sector, specifically the Artificial Intelligence subsector, and the occupation Machine Learning Engineer. Along with the occupation, the Sunburst-type chart shows the related KSAs. A bigger-sized skill represents a more important skill related to the occupation. This is a visual result aid for the public that seeks to know the KSAs that are in trend to seek to cover the occupation in the best way, as well as those who are in the occupation and want to perform upskilling or reskilling to be up-to-date with what the current industry requires to cover the occupation.

The proposed chart has proven to be effective in aiding in the visualization of structures similar to the one found in this work. The proposed chart types can be adjusted to the number of terms and levels desired in the taxonomy representation.

These types of KSA taxonomies can provide a wealth of applications across a variety of fields, thanks to their versatility and value as a tool for aligning skills with industry needs. In the technology sector, the taxonomy can be customized to identify emerging competencies such as machine learning operations, artificial intelligence, cloud infrastructure management, and cybersecurity, allowing organizations to refine job profiles and plan upskilling initiatives. In the healthcare sector, the taxonomy can help define roles that involve advancements such as telemedicine, AI-powered diagnostics, and patient data management, enabling targeted training programs that keep pace with technological innovations. For the education sector, this taxonomy offers a framework for designing adaptable curricula that integrate real-time skill demand data, ensuring graduates are workforce-ready. In the automotive sector, the taxonomy can be tailored to map skills related to new occupations related to electric vehicles, autonomous driving systems, and advanced manufacturing processes, providing automakers with tools to adapt workforce training to emerging technologies and sustainability trends. Additionally, industries such as manufacturing and renewable energy can benefit from customized taxonomies that track changes in skills related to automation, IoT, and green technologies. By leveraging its dynamic, real-time updating capabilities, this taxonomy serves as a strategic resource for workforce planning, policymaking, and customized career development, ensuring relevance in rapidly evolving domains.

## 5 Discussion

Our Relation Extraction model displayed a promising trajectory of learning and optimization, as evidenced by the gradual decrease in loss values and the improvement in precision and F1-scores over 10,000 training epochs. This progression indicates the model's growing accuracy and efficiency in identifying and categorizing relationships between entities, a critical component in the field of NLP. Particularly noteworthy is the model's consistently high recall with the NER model, suggesting a good ability to identify relevant entities. However, this may also indicate a potential inclination toward recall, raising concerns about the precision and the possible increase in false-positive rates. This aspect underscores the necessity for a more nuanced approach to balance precision and recall, ensuring a holistic and reliable performance of the model.

This work is at the state-of-the-art in the construction of dynamic taxonomies, setting itself apart from existing studies in several key respects. By taking advantage of the power of NLP through PDF parsing, text extraction, tokenization, and lemmatization, our approach transcends the static confines of traditional taxonomies. Unlike conventional systems, our dynamic taxonomy not only identifies current competencies but, more importantly, anticipates and classifies the evolving skill sets that will shape the workforce of the future. The way that visualization changes impacts how it can be interpreted, both immediately and over time (Cottam et al., 2012).

This work reveals some significant practical implications in its potential application by educational institutions and workforce organizations. Educational institutions could use the dynamic taxonomy to align their curricula with current skill demands. By incorporating the taxonomy's predictive capabilities, universities and training providers can design programs that seek to address emerging industry needs, ensuring graduates are equipped with knowledge, skills, and attitudes relevant to the future. For example, the taxonomy could inform course offerings in fields such as data science, renewable energy, and healthcare, where skill requirements are rapidly evolving.

Similarly, workforce development organizations can integrate this taxonomy into their training initiatives to enable specific upskilling and reskilling. By identifying the most critical knowledge, skills, and attitudes for specific sectors, such as technology, automotive, and manufacturing, organizations can create customized training programs that close skills gaps and improve employee readiness for industry changes. Policymakers could also use this tool to support workforce planning strategies, ensuring alignment between labor market demands and educational policies.

Compared to previous research, our work adds a predictive dimension to taxonomy construction, offering a future perspective on workforce competencies. The integration of NLP techniques helps to understand the textual content, allowing us to extract insights that go beyond the immediate context. This potential for change transforms our dynamic taxonomy into a proactive tool for organizations seeking to navigate the evolving scenario of skills requirements.

Using Named Entity Recognition techniques proved beneficial for identifying terms of interest, such as occupations, skills, and knowledge, in unstructured text. The model demonstrated adaptability to different domains and case studies without major modifications, showcasing its versatility. In terms of complexity, the NER component exhibited more ease in finding and annotating skills compared to occupations, possibly influenced by token summarization for multi-word terms.

The Relation Extraction component outperformed the initial NER, achieving an F1 score of 82.2%. This success was attributed to a well-described training corpus that effectively captured relations between occupations and required skills. While NER accuracy rates suggest the model's potential for novel entity representations, a thorough discussion on the practical significance of these rates in real-world scenarios is essential for a comprehensive evaluation.

The implementation of this integrated approach reduces the time required for skills classification taxonomy analysis. In contrast to traditional methods, our strategy, as outlined in the objectives, streamlines the process. The capability of extracting insight from unstructured texts is an indicative of the potential impact on accelerating insights and decision-making in the realm of skills taxonomy.

Highlighting the model's capacity for generalization, especially with limited training samples, is crucial for understanding its effectiveness with an expanded dataset. Additionally, evaluating the consequences of misclassifications in practical settings provides insights into the model's reliability. Addressing variance in entity recognition across diverse documents enhances our understanding of the model's adaptability to different text formats and real-world data variations.

However, it is crucial to realize certain limitations. Although exhaustive, the scope of the study is limited by the number of documents analyzed. Extending this approach to larger data sets is a challenge that can be considered in future research. In addition, the effectiveness of dynamic taxonomy may vary across sectors and domains, justifying careful examination in various applications.

## 6 Conclusions and future work

This study has marked a significant achievement in establishing a system for identifying skills and relationships, with the help of NLP tools from two models: NER and RE. The implementation of these models, trained and optimized over 10,000 epochs, has culminated in a system that not only identifies skills accurately but also captures the complex relationships between them.

The evolution of loss metrics throughout training reflects the effectiveness of the model's learning process. The accuracy and F1-score results in relation extraction are particularly remarkable, with a significant increase from modest initial values to impressive figures at the end of training.

In developing this system, we have not only succeeded in building a solid framework for skill and relationship identification, but we have also laid the groundwork for future improvements. Automation of the process to keep the taxonomy dynamic, continuous expansion of the corpus from key sources, and constant improvement of the model are essential steps toward a more complete and adaptable system.

Looking ahead, this research lays the groundwork for several promising avenues in the development and application of dynamic taxonomies. One important area of future exploration is the expansion of the document corpus to accommodate a larger and more diverse dataset.

In addition, the integration of advanced machine learning models offers the potential to refine the predictive capability of the taxonomy. By incorporating models capable of discerning subtle language changes, the taxonomy can better anticipate emerging competencies.

An adaptive taxonomy ensures that it remains relevant and accurate in dynamic environments, reflecting the constantly evolving nature of workforce competencies. Despite these limitations, our work opens avenues for future research in the construction of dynamic taxonomies. The ability to not only react to current competencies but also to predict future trends provides a strategic advantage in planning. This approach not only improves our understanding of current competencies but also provides insight into the evolving demands of the industry in the future.

This job successfully demonstrates the integration of advanced NLP techniques, including Named Entity Recognition and Relation Extraction, with custom sub-word embeddings and lexicons, within the proposed architecture. The dynamic skills taxonomy crafted through this integrated approach not only reveals insightful relationships between concepts but also effectively categorizes emerging skill requirements in the ever-evolving job market. The adaptability and user-friendliness of the resulting taxonomy provide a valuable resource for navigating the fluid nature of work and preparing for a sustainable future workforce.

Moving ahead, the focus of future work is on validating taxonomy relationships through various evaluation techniques, including expert reviews and an ongoing survey. The survey aims to efficiently gather extensive insights from experts, contributing to the refinement of the dynamic skills taxonomy. This ensures its continuous relevance in the evolving labor market. Additionally, the efforts made in this work contribute to completing a predictive model for understanding future skill requirements in current and future occupations.

This approach not only reveals insightful relationships between concepts but also effectively categorizes emerging skill requirements in the ever-evolving job market. The resulting taxonomy is adaptable and user-friendly, providing a valuable resource for navigating the fluid nature of work and preparing for a sustainable future workforce. Furthermore, the implementation of this approach significantly reduces the time required for skills classification taxonomy analysis compared to traditional methods, expediting decision-making in the skills taxonomy domain.

This work sets the stage for future work, emphasizing the validation of taxonomy relationships and suggesting exploration into Generative AI. Collaborative efforts with Generative AI hold promise for refining and augmenting the predictive model. Additionally, future research may explore novel approaches to incorporate semantic context, mitigating challenges with ambiguous terms and contributing to a nuanced understanding of the dynamic interplay between occupations, skills, and knowledge. The development of dynamic taxonomy visualizations and the nuanced exploration of term evolution over time emerge as captivating avenues for future research, offering comprehensive insights into the intricate relationships within the ever-changing world of work.

## Data Availability

The raw data supporting the conclusions of this article will be made available by the authors upon request.
